# Direct Excision of Malar Bags: Back to the Basics

**DOI:** 10.1007/s00266-024-04411-5

**Published:** 2024-10-01

**Authors:** Giovanni Botti, Chiara Botti, Mariachiara Fabbri, Marta Mariani, Vittoria Murone, Benedetta Scucchi, Pietro Luciano Serra

**Affiliations:** 1 Plastic Surgeon in Private Practice at Villa Bella Clinic, Via Europa 55, 25087 Salò, Italy; 2 Residency Program in Plastic Surgery, Università Cattolica del “Sacro Cuore”, Largo A. Gemelli 8, 00168 Rome, Italy; 3 Residency Program in Plastic Surgery, Azienda Ospedaliera Universitaria Federico II di Napoli, Via Sergio Pansini 5, 80131 Napoli, Italy; 4 Residency Program in Plastic Surgery, Azienda Ospedaliera Universitaria di Padova, Via Niccolò giustiniani 2, 35121 Padova, Italy; 5 Residency Program in Plastic Surgery, Plastic Surgery Unit, Department of Medical Surgical and Experimental Sciences, University of Sassari, Sassari University Hospital Trust, 07100 Sassari, Italy

**Keywords:** Periorbital rejuvenation, Malar bags, Direct excision, Festoons, Aesthetic outcomes

## Abstract

**Introduction:**

The pursuit of periorbital rejuvenation to counteract signs of aging is a focal point in cosmetic surgery, with eyelid surgery being a prominent choice among patients. Addressing inferior blepharoplasty, especially in cases involving chronic malar edema, malar mounds, and festoons, presents unique challenges. The terminology and classification of these conditions vary, hindering effective management. In this study, we use the term “malar bags” to encompass the spectrum of fluid-associated mounds over the malar eminence.

**Objectives:**

This study aims to demonstrate the effectiveness of direct excision as a surgical choice for treating the aesthetic concerns associated with malar bags.

**Materials and Methods:**

A retrospective study was conducted on 53 patients who underwent direct excision of malar bags between 2013 and 2023 at our clinic.

**Results:**

No major complications were encountered, overall high level of satisfaction for both patients and surgeons.

**Conclusion:**

Direct excision of malar bags proved to be a safe and effective technique, yielding satisfactory results in terms of both aesthetic outcomes and patient satisfaction.

**Level of Evidence III:**

This journal requires that authors assign a level of evidence to each article. For a full description of these Evidence-Based Medicine ratings, please refer to the Table of Contents or the online Instructions to Authors www.springer.com/00266.

## Introduction

The early signs of aging manifest prominently in periorbital changes, emphasizing the crucial need for periorbital rejuvenation to restore a graceful appearance. Eyelid surgery stands out as one of the most sought-after aesthetic procedures, evident in the approximately 33.5 thousand eyelid surgeries performed in Italy in 2022 [1]. This procedure reigns supreme among face and head cosmetic surgeries in the country. However, addressing inferior blepharoplasty poses unique challenges, particularly in patients with chronic malar edema, malar mounds, and festoons on the lower eyelid and cheek.

Achieving a natural and aesthetically pleasing lower eyelids requires intricate restoration of the youthful lid-cheek junction and malar eminence. Yet, the spectrum from malar edema to festoons presents an ongoing challenge when dealing with these enigmatic “malar bags.” Managing malar bags proves problematic due to diverse pathophysiology, inconsistent terminology, and variable severity concerning patients’ malar edema, mounds, and festoons.

Kpodzo and colleagues propose a classification using three specific terms: malar edema, malar mound, and festoons. Malar edema denotes the presence of fluid over the malar eminence, while malar mound refers to a soft tissue prominence, typically involving the orbicularis muscle or fat, over the same area. Festoons are described as “cascading hammocks of lax skin and orbicularis muscle” hanging between the medial and lateral canthi [2]. However, given that these terms could also represent different points along the spectrum of the same underlying pathophysiological condition, in this article we will use the term “malar bags” to encompass the entire range of fluid-skin-muscle-fat-associated mounds over the malar eminence.

Malar bags manifest as a triangular-shaped fold below the ORL and the lateral inferior orbital rim, often surpassing medially the midpupillary line or even spanning from medial canthus to lateral canthus, also surpassing the latter to extend even further laterally—hence the term “malar.”

Orbicularis weakness or attenuation plays a central role in their development, posing a challenging entity despite advancements in lower lid blepharoplasty.

**Fig. 1:**
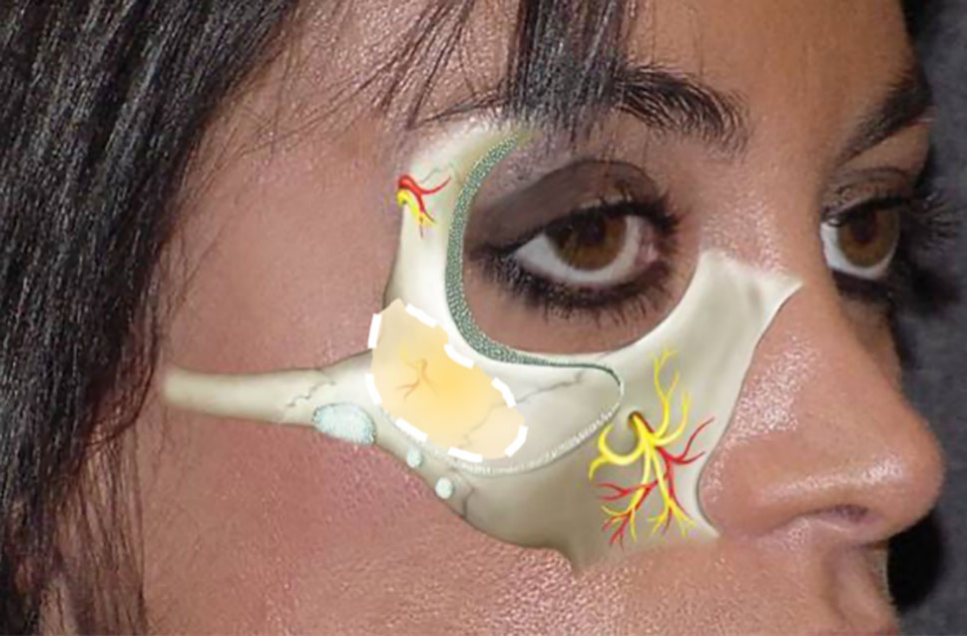
Picture showing the roof and floor of malar bags, ORL and ZCL, respectively

A thorough grasp of anatomy is essential for the accurate treatment of these aesthetic conditions. As delineated by Furnas [3], our understanding indicates that malar bags occupy a space delimited superiorly by the orbicularis retaining ligament (ORL) and inferiorly by the midcheek fold, specifically Pessa and Garza’s malar septum, situated 2.5 to 3 cm below the lateral canthus [4]. Malar bags always form above the chain of zygomatic ligaments because these ligaments are very robust. By connecting the skin to the periosteum, they limit the skin’s ability to move along the lower edge of the zygomatic bone, which, with aging or lymphatic stasis, creates a fold (Fig.1).

The clinicopathology of malar bags remains poorly understood, manifesting as tissues descend in a redundant fashion over the malar eminence. While often associated with aging, these conditions can also occur early in life due to a combination of factors including tissue laxity (skin and orbicularis oculi), weakened ORL, impaired lymphatic drainage and compromised attachment of the inferiorly located zygomaticocutaneous ligament (ZCL) [5, 6] .

Surgeons must assess orbicularis oculi muscle and skin laxity through the pinch and squinch test [3] to determine the composition of malar bags, guiding appropriate surgical interventions such as fat-muscle-skin excision or suspension.

The range of advocated techniques signals the variability in outcomes, highlighting the challenge of any single method consistently delivering good and satisfactory results.

Surgical interventions remain the “gold standard” for severe or persistent malar bags. Over the years, various surgical treatments have been proposed to address this condition; however, there is still no universally accepted surgical approach suitable for all patients.

In this article, we aim to share our extensive experience with the direct excision of malar bags, focusing on the technique employed and the long-lasting results achieved. We will also discuss the possibility of using an extended blepharoplasty, which can also offer excellent results in these cases, if performed with care.

## Materials and Methods

This retrospective study encompassed a cohort of 53 patients, including both male and female individuals, who underwent direct excision of malar bags within the timeframe spanning from 2013 to 2023 at our clinic under the care of the senior authors. The mean age of the cohort was 51 years. Among the participants, 60% exhibited pure festoons characterized by skin and orbicularis oculi muscle involvement, while the remaining 40% presented with malar bags demonstrating a notable adipose component. Exclusion criteria encompassed patients primarily afflicted with malar edema, whose condition was initially managed through non-surgical techniques. Prior to intervention, all patients received comprehensive explanations delineating the proposed surgical technique, anticipated temporary visibility of resultant scarring, and were required to provide informed consent, which was duly documented. Ethical considerations were meticulously observed in accordance with the principles delineated in the Declaration of Helsinki.

The surgical outcome was evaluated based on follow-up examinations and photographic documentation. Satisfaction was assessed informally during follow-up visits through direct patient feedback and observation of clinical outcomes. Notably, none of the patients required revision surgery, and all patients expressed verbal satisfaction with the results during these visits. Objective measurements of scar visibility, eyelid position, and symmetry were also performed during follow-up visits at 3 months, 6 months, and 1 year postoperatively.

### Surgical Technique

**Fig. 2:**
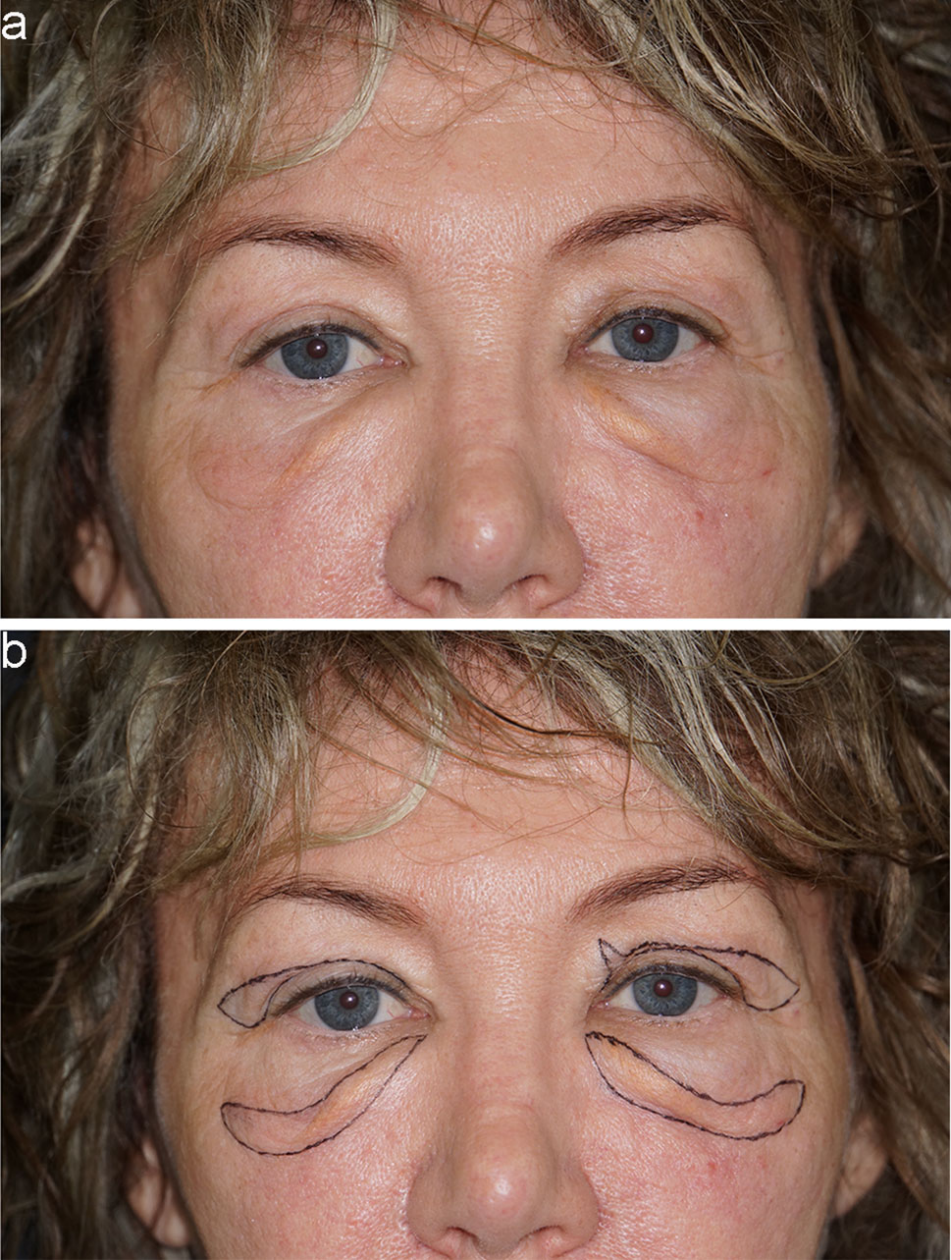
A 62-year-old female patient. **a** Preoperative photo showing prominent malar bags. **b** Preoperative markings based on the deformity

Preoperatively, the targeted area is carefully marked based on the specific deformity (Fig. 2). After local anesthetic infiltration (mepivacaine 2%, adrenaline 1:100.000), the surgical field is meticulously prepared in a sterile manner. Using a no. 15 blade, an incision is made following the preoperative markings, followed by the excision of the deformity and meticulous hemostasis achieved with monopolar cautery.

**Fig. 3:**
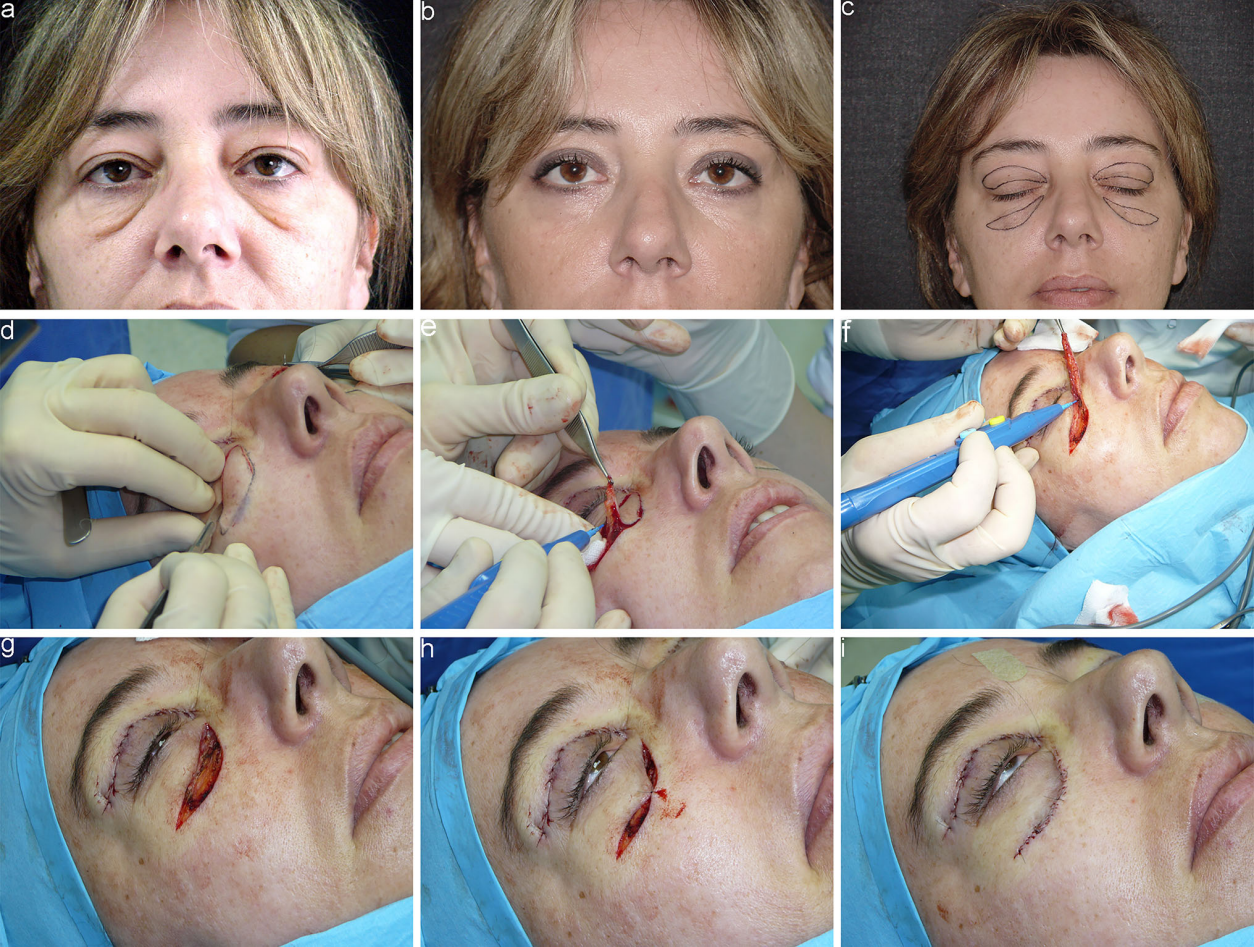
A 50-year-old female patient. **a** Preoperative frontal view showing prominent malar bags. **b** Postoperative frontal view at 1 year. **c** Preoperative markings. **d-h** Intraoperative passages showing removal of only excessive skin and malar fat, leaving OOM intact. **i** Immediate postoperative result showing external running suture. Upper blepharoplasty was carried out as well.

**Fig. 4:**
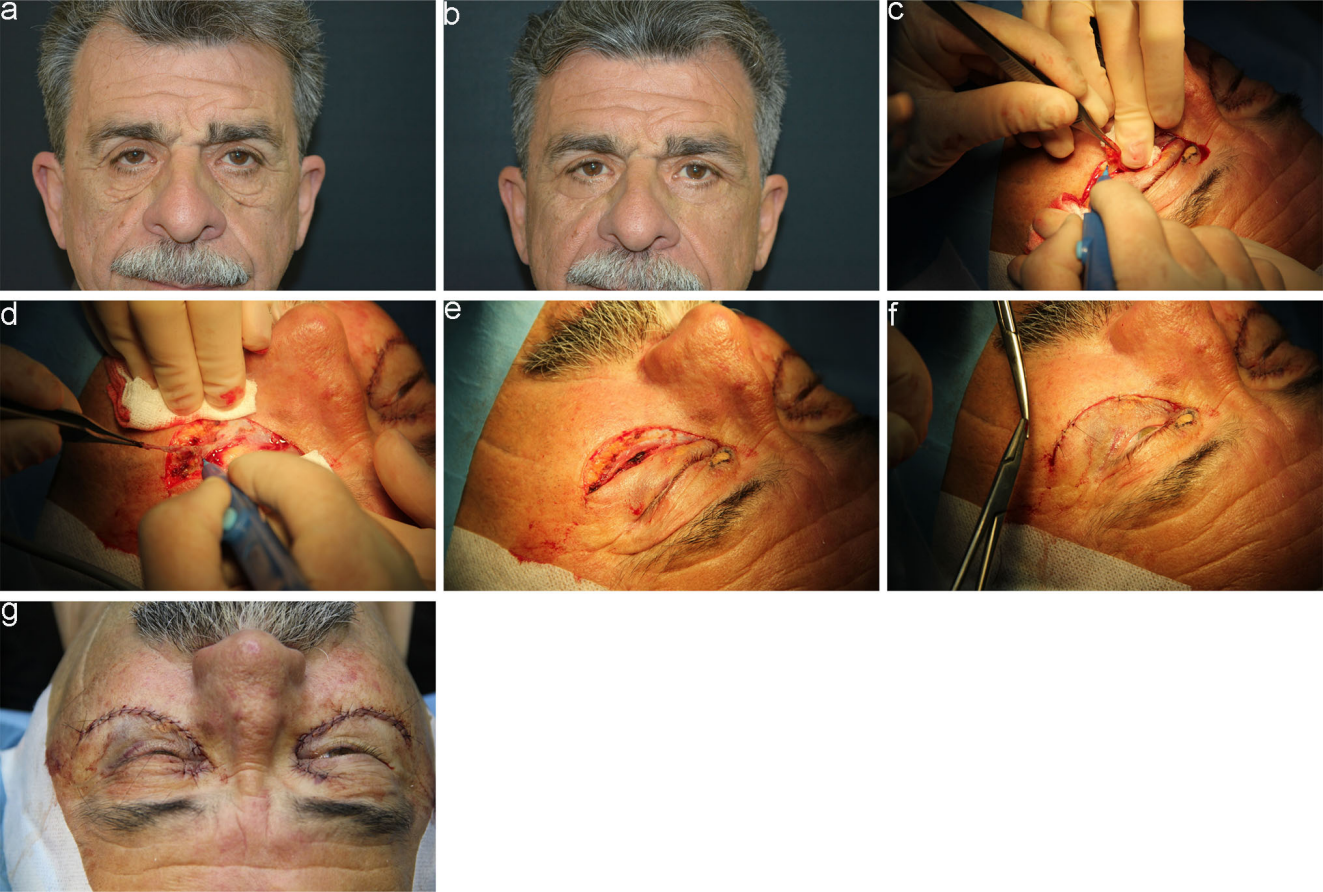
A 70-year-old male patient **a** Preoperative frontal view showing prominent malar bags. **b** Postoperative frontal view at 1 month. **c-f** Intraoperative passages showing removal of excessive skin and malar fat and a strip of OOM. **g** Immediate postoperative result showing external running suture

The extent of tissue removal is determined by the composition of the malar bags: cases involving excess skin and laxity, with or without malar fat, may involve halting resection at the orbicularis oculi muscle underneath (Fig. 3). However, in most instances, there will also be excess laxity of the orbicularis oculi muscle, which may necessitate its partial removal, sometimes also extending to the underlying adipose component. To be more precise, in elderly patients undergoing this surgery, the orbicularis oculi muscle is typically lax and tends to cover nearly all the malar bone, causing a weighted appearance in the area. Hence, its removal constitutes a crucial step in this procedure, while always ensuring preservation of the underlying lip elevator muscles (Fig. 4).

The degree of superior subcutaneous undermining is carefully regulated and should be conservative. While there is a desire to position the scar along the eyelid margin to minimize its visibility, this must be balanced against the potential risk of eyelid margin descent.

The inferior subcutaneous undermining may be extended by approximately 5 mm to a maximum of 10 mm in specific cases, facilitating the advancement of the lower flap while preventing downward displacement of the lower eyelid. In instances where challenges arise in aligning the two flaps, leading to a heightened risk of inferior displacement of the lower eyelid, securing the lower flap to the periosteum of the zygomatic bone with 4/0 Vicryl can evenly distribute tension across deeper planes. However, it is essential to note that this approach should be reserved for situations where absolutely necessary, as the periosteum in this region is not notably dense, and the procedure carries inherent risks, including the rare possibility of facial nerve injury.

Precise approximation of margins is achieved with subcutaneous sutures using 5/0 Vicryl, followed by a final continuous running partially everting suture using Nylon 6/0. Particular care is taken to avoid any tension on the lower eyelid.

Postoperative care includes applying paper patches covered by a foam pad, secured to the skin with additional paper tapes, to provide compression during the initial days following surgery. Outer running sutures are removed on the 4th postoperative day, and paper patches are applied for the subsequent week. After the removal of paper patches, patients are instructed to apply silicone patches at home and during the night for 3 months, avoid sun exposure for at least a year and apply SPF 50+ sunscreen daily.

## Results

**Fig. 5:**
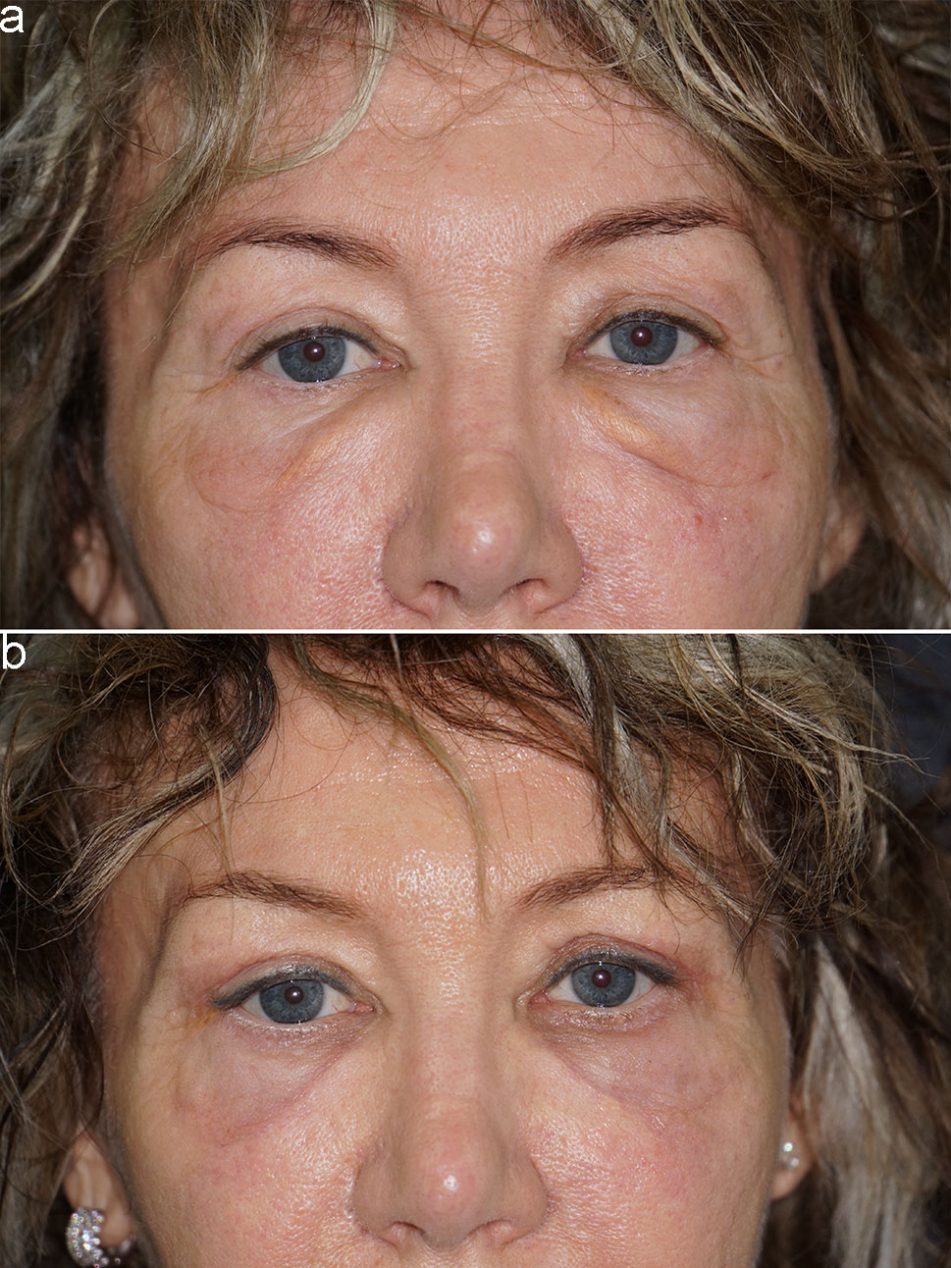
A 62-year-old female patient. **a** Preoperative frontal view showing prominent malar bags. **b** Postoperative frontal view at 1 month

**Fig. 6:**
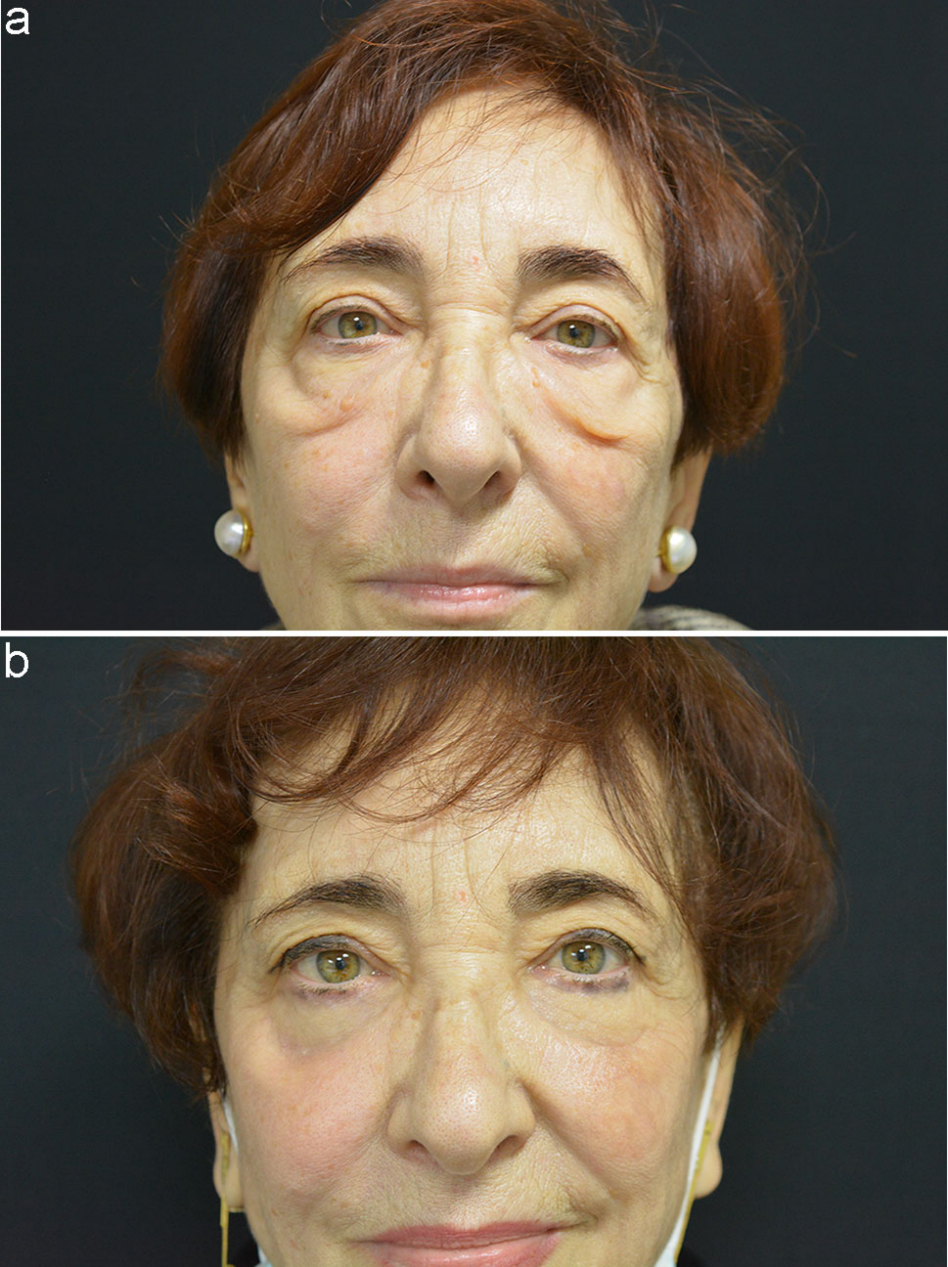
A 66-year-old female patient. **a** Preoperative frontal view showing prominent malar bags. **b** Postoperative frontal view at 1 year

The average follow-up period was 2 years. No major complications were observed in any of the patients in this series. Three out of 53 patients developed transient slight lower eyelid dislocation, which resolved spontaneously, while 2 out of 53 patients developed a slightly visible scar. None of the patients in the study required reoperation, scars were almost invisible, and overall satisfaction was very high for both patients and surgeons (Figs. 5–6).

## Discussion

Various surgical interventions have been employed over the years to address malar bags, yielding different levels of success.

Rosenberg [7] introduced the use of superficial suction lipectomy for treating malar bags, targeting the subcutaneous fat between the dermis and orbicularis muscle. However, while effective for fat removal, this technique does not address orbicularis muscle, skin excess, or lid edema, and it carries risks such as irregularities of the skin surface and long-lasting swelling.

Liapakis and Paschalis [8] proposed a combination of liposuction and orbicularis oculi suspension for malar bags treatment while Asaadi [9] emphasized the importance of lipectomy in surgical treatments for congenital festoons, as the etiology differs from acquired festoons.

Furnas [3] in 1978 followed by Rees and Tabbal [10] in 1981 described the use the skin-muscle flap procedure, an extended blepharoplasty that includes lifting both the skin and the orbicularis oculi muscle. This approach, designed to address under-eye bags, involves excising excess skin and muscle tissue and then redraping the remaining flap across the festoon. However, it does not address the underlying ligaments or the primary pathophysiology, such as ligamentous laxity or tissue ptosis, which are often key factors in aging-related changes around the eyes. The extended lower blepharoplasty involves extending the dissection of the skin-muscle flap used in standard lower eyelid blepharoplasty to a level below the infraorbital rim. This technique is particularly useful in managing infraorbital or malar bags, but it is much more demanding both in terms of indispensable technical skill and predictability of a good outcome.

In general, both direct excision and extended blepharoplasty may serve as viable options for addressing the same condition; however, the former typically carries a diminished risk of eyelid lowering and displacement.

Another described approach is the subperiosteal midface lift, which involves the en bloc upper displacement of all soft tissue between the skin and the bone.

Various surgical techniques, such as the video-assisted endoscopic subperiosteal vertical upper-midface lift (SUM-lift) [11], the concentric malar lift [12], and the triple-layer midface lift [13], have been employed for midface elevation and festoon correction. Most of these techniques involve addressing the lower eyelid through subciliary incisions followed by a subperiosteal dissection and a suspension of midface soft tissues to the deep temporal fascia and/or to the orbital rim. Anastassov and St Hilaire’s midface lift technique [14], Owsley and Zweifler’s emphasis on midface lifting for various concerns [15], and Krakauer and colleagues’ subperiosteal midface lift [16] further illustrate the versatility of these approaches in treating malar descent, festoons, and related aesthetic issues. However, severe complications like lower lid margin displacement, and milder ones like hematoma formation, and postoperative long-lasting edema must be taken into account when opting for these approaches. Moreover, a primary concern regarding surgeries involving deep dissection on the bone surface is the potential for elevating all structures, including the defect, without effectively addressing its elimination. When employing subperiosteal lifting as a method to address malar bags and festoons, it is crucial to intervene at two levels: performing deep subperiosteal dissection and superficial sub-orbicularis oculi muscle dissection [17].

The direct excision of malar bags is not a recent innovation. It was first documented in 1995 by Netscher et al. [18] and subsequently reported by Bellinvia et al. [19] in 2010 and Einan-Lifshitz et al. [20] in 2012. This approach is typically chosen when skin excess and/or laxity plays a significant role in the issue, but it can be adopted in cases involving orbicularis laxity, redundancy, or general soft tissue ptosis. Opting for direct excision can help mitigate specific risks, such as ectropion, scleral show or lagophthalmos, which are much more frequently associated with some alternative techniques. This procedure is particularly well-suited for older patients with deep wrinkles and crepe-like skin, as the resulting scar is likely to be less noticeable in this demographic. It is a quick and straightforward procedure designed to directly tackle the issue of malar bags. However, we must contend with certain side effects, including visible scars and potential lid retraction. To minimize the risk of unsightly scarring, it would be appropriate to place the scar at the edge of the finer eyelid skin rather than in the thicker cheek skin. Furthermore, precise margin approximation and suturing are essential.

This study’s retrospective design and single-clinic setting introduce limitations, including potential patient selection bias and the absence of a control group, which limit the generalizability of the results. The lack of a formalized patient satisfaction survey also poses challenges to the objectivity and consistency of outcome assessments. Furthermore, the relatively small sample size may affect the statistical power, potentially influencing the reported rates of complications and patient satisfaction. Despite these limitations, the study provides valuable insights into the efficacy and safety of the direct excision technique, laying the groundwork for future prospective studies with larger cohorts and standardized protocols.

## Conclusion

The treatment of malar bags remains a persistently challenging issue for both patients and surgeons. Despite the development of numerous techniques over the years to address this condition, a consistent consensus on the most effective procedure is still lacking. As has often been observed in the history of plastic surgery, simplicity is frequently the key to success. We strongly endorse this philosophy. Consequently, after experimenting with various surgical approaches to treat this specific condition, we returned, when possible, to the fundamentals: a straightforward, swift, and safe procedure. This approach avoids significant complications while delivering excellent aesthetic results and achieving a high rate of patient satisfaction.
